# Gold Nanoparticles dotted Reduction Graphene Oxide Nanocomposite Based Electrochemical Aptasensor for Selective, Rapid, Sensitive and Congener-Specific PCB77 Detection

**DOI:** 10.1038/s41598-017-05352-7

**Published:** 2017-07-12

**Authors:** Lidong Wu, Xianbo Lu, Xiaochen Fu, Lingxia Wu, Huan Liu

**Affiliations:** 1 Key Laboratory of Control of Quality and Safety for Aquatic Products, Minisrty of Agriculture, Chinese Academy of Fishery Sciences, Beijing, 100141 China; 2 Key Laboratory of Separation Science for Analytical Chemistry, Dalian Institute of Chemical Physics, Chinese Academy of Sciences, Dalian, 116023 China

## Abstract

Gold nanoparticles (AuNP) dotted reduction graphene oxide (RGO-AuNP) is used as a platform for an aptamer biosensor to selectively detect 3,3′4,4′-polychlorinated biphenyls (PCB77). By anchoring aptamers onto the binding sites of RGO-AuNP and making use of the synergy effect of RGO and AuNP, the RGO-AuNP based biosensor exhibits superior analytical performances to AuNP based biosensor in terms of sensitivity and repeatability. The sensitivity of RGO-AuNP based aptamers (RGO-AuNP-Ap) biosensor (226.8 μA cm^−2^) is nearly two times higher than that of Au based biosensors (AuNP-Ap/Au electrode, 147.2 μA cm^−2^). The RGO-AuNP-Ap/Au biosensor demonstrated a linear response for PCB77 concentrations between 1 pg L^−1^ and 10 μg L^−1^, with a low limit of detection (LOD) of 0.1 pg L^−1^. The superb LOD satisfies the exposure thresholds (uncontaminated water < 0.1 ng L^−1^) set out by International Agency for Research on Cancer (IARC) and the Environmental Protection Agency (EPA). The proposed biosensor can be a powerful tool for rapid, sensitive and selective detection of PCBs on site.

## Introduction

Polychlorinated biphenyls (PCBs) are a family of 209 chemically related compounds that were widely used in a number of industrial applications. PCBs produce a number of adverse health effects including immunotoxicity, neurotoxicity, reproductive toxicity and carcinogenesis^1–3^. Two major structural classes of PCBs include the co-planar PCBs, which include several ‘dioxin-like’ PCBs such as 3,3′,4,4′-polychlorinated biphenyl (PCB77)^4, 5^ and non-coplanar derivatives, which have been widely dispersed into environment. PCB77 is one of the most toxic PCB congeners but is present at comparatively low concentrations in the environment^5^. Because of the high toxic equivalency factor (TEF) of PCB77 and the concentration correlation of PCB77 with other PCBs, the concentration of PCB77 can also be used as an indicator for the pollution level of PCB congeners. Rapid, sensitive, cost-effective, portable and on-site screening system for PCBs is urgently needed to ensure food safety and is the very important first step in environmental risk evaluations.

Traditionally, PCB congeners are detected by Gas Chromatography-Mass Spectrometer (GC-MS)^6^. These traditional techniques possess good precision and high resolution but it requires complicated time-consuming sample pre-treatment processes, professional technician, and expensive instruments. Therefore, there is an urgent demand to develop a cost-effective, simple and rapid method for determining PCBs in environmental sample. The biosensor method can be an alternative tool to solve the above problems. Conventional biosensors which are based on DNA as recognition elements were developed to cost-effective and portable detection of PCBs. However, the selectivity and detection limit of those reported biosensor^7, 8^ is not satisfactory for their potential applications. Therefore, there is a critical need to build a more selective method to complement the disadvantages of the conventional biosensors.

Aptamer is single-stranded DNA or RNA with a high affinity for target and referred to as “chemical antibodies”, and it can give a better choice to current approaches. Because of its unique three-dimensional structures, aptamers can selectively recognize a target or a family of targets. Furthermore, aptamers are cost-effective, easily prepared, reproducible, animal-friendly and nonimmunogenic^9^. Up to date, few studies have been reported for the selective detection of PCBs using an aptamer biosensor because PCBs are stable unreactive chemical compounds^10^.

Biosensing materials play a vital role in the development of aptamer biosensor^11^. Designing the superior biosensing material is the core technology of aptamer biosensor for improving long-stability *in vitro*, limit of detection (LOD) and sensitivity. Graphene oxide has sparked increasing attention as novel two-dimension nanomaterial because of its excellent chemical and thermal stability and high specific surface area. Reduced graphene oxide possesses better electric conductivity than graphene oxide. The combination of gold nanoparticles (AuNPs) with chemically reduced graphene oxide in aqueous solutions can create unique hydrophilic and multi-active sites nanocomposites which provide more self-assemble sites to graft the DNA aptamer segment.

In this work, an electrochemical PCB77-binding DNA biosensor based on RGO-AuNP nanocomposites is established for rapid detecting of PCB77 with high selection and sensitivity, and this work provides significantly better sensitivity and limit of detection (LOD) than previously reported^12^. PCB77-binding DNA aptamer isolated by SELEX (systematic evolution of ligands by exponential enrichment) and electroactive ferrocene (Fc) binding with 3′-DNA aptamer are used as biorecognition element and signal amplification molecules to determine PCB77 compounds with high selection and sensitivity, respectively. The RGO-AuNP-Aptamer/Au biosensor demonstrates a linear response with PCB77 concentrations ranging from 1 pg L^−1^ to 10 μg L^−1^, and its limit of detection (LOD) achieves 0.1 pg L^−1^. It is supposed to be a potential tool for selectively rapid determining of PCB77 on site.

## Results and Discussion

### Physical Characterization of RGO and RGO-AuNP

**Figure 1:**
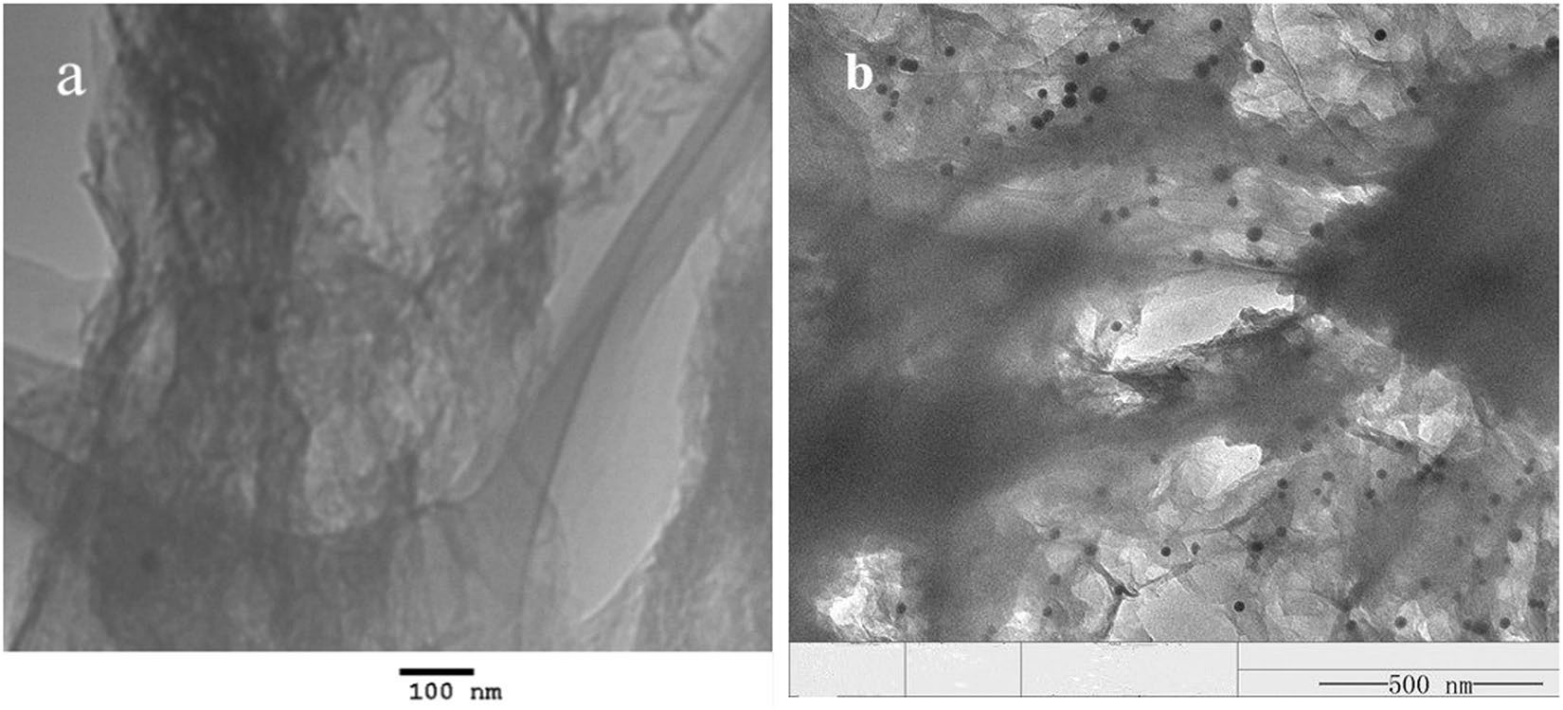
TEM images of reduction graphene oxide (**a**) and reduction graphene oxide-gold nanocomposites (**b**).

The structure and morphology of the obtained RGO and RGO-AuNP was characterized by TEM and the Fig. 1 displays their representative TEM images. As shown in Fig. 1, the gold nanoparticles are monodispersed onto the surface of RGO, with the mean particle size being around 20 nm. This is possibly due to that the RGO provides strong driving force for self-assembly of the AuNPs and connects with AuNPs by electrostatic attraction. This nanocomposite is more controllable than previous reported nanocomposite^13^.

The RGO-AuNP nanocomposites were characterized by UV-Vis spectroscopy as shown in Figure S1. The figure demonstrates two separate peaks around 260 and 520 nm for the nanocomposites. The peak around 260 nm corresponds to the π-π* transition of the aromatic C-C ring in RGO^12^ and the one at 520 nm attributes to the typical surface-plasmon resonance band of gold nanoparticles (about 20 nm)^14^. The presence of the bands at 260 and 520 nm indicates that the surface of RGO has been successfully modified by gold nanoparticle. This has also been evidenced by the photographs (Figure S2) via dispersions of the same concentration (0.4 mg ml^−1^) of AuNPs, RGO and RGO-AuNPs in aqueous solution. As shown in Figure S2A, the color of AuNPs is wine red which ascribes to the particle size of AuNPs (20 nm)^15^. As AuNPs combined with RGO, the color of RGO dispersion changes from light brown (Figure S2B) to black (Figure S2C)^16^. This also reflects that the gold nanoparticles are monodispersed onto the surface of RGO.

The surface charge of RGO and gold nanoparticles were tested by zeta potential center (Zetasizer, Malvern, UK). Generally, particle size with zeta potential in the range −30 to +30 mV are considered stable due to electrostatic repulsion. The zeta potential of RGO and gold nanoparticles are −46.8 and −32.3 mV, respectively. The X-ray photoelectron spectroscopy (XPS, Figure S3) was also used to confirm whether the RGO-AuNPs nanocomposite has been obtained or not. XPS analysis indicated the presence of gold nanoparticles and carbon in RGO-AuNPs.

### Monitoring the Immobilization of the RGO-AuNP-Ap/Au Biosensor

**Figure 2:**
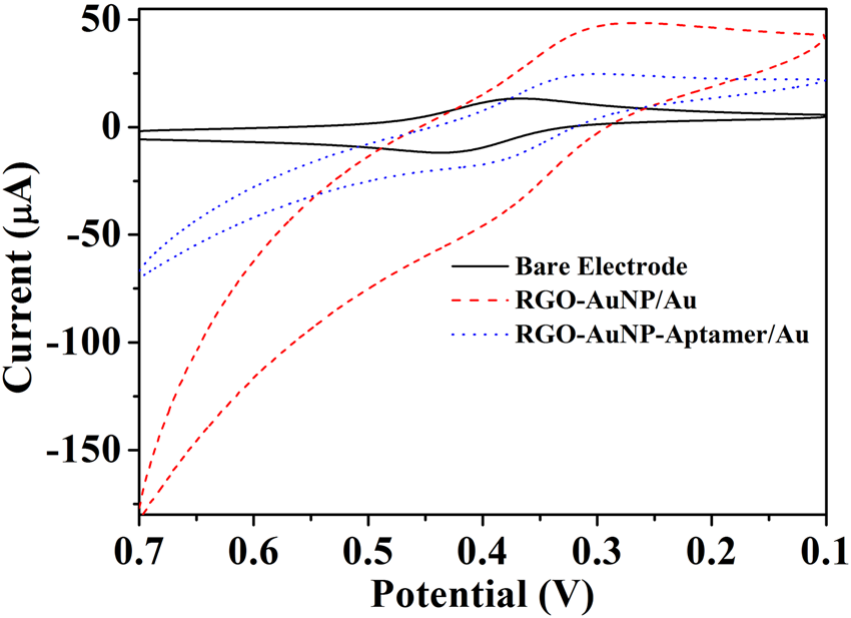
Cyclic voltammograms of the different Au electrodes in 5 mmol L^−1^ [Fe(CN)_6_]^3−/4−^ (**a**) Bare Au electrode (black line, solid line), (**b**) RGO-AuNP/Au electrode (red line, dashed line) and (**c**) RGO-AuNP-Aptamer/Au electrode (blue line, dotted line).

In this study, the modification process of the Au electrode was monitored by cyclic voltammetry (CV) in a [Fe(CN)_6_]^4−/3−^ (FC) solution. Figure 2 shows the CV signals of the bare Au electrode, RGO-AuNP/Au and RGO-AuNP-Ap/Au electrode (curve a, curve b and curve c) in a [Fe(CN)_6_]^4−/3−^ solution. The peak separation between the cathodic and anodic peaks ascribes to the [Fe(CN)_6_]^4−/3−^ transformation on the bare electrode (curve a) which is around 65 mV. It indicates that the electrochemical redox process is a reversible one-electron redox process. The response signal of [Fe(CN)_6_]^4−/3−^ on the RGO-AuNP/Au electrode (curve b) increases remarkably, which is primarily due to the RGO-AuNPs nanocomposites on the surface of the gold electrode. After the aptamer immobilized onto the RGO-AuNPs/Au electrode, the response signal of [Fe(CN)_6_]^4−/3−^ decreases obviously. This is mainly because the negative charged phosphate skeleton of the aptamer produces the repulsive force to make the signal molecules ([Fe(CN)_6_]^4−/3−^) away from the surface of electrode and the ferrocene modified 3′-aptamer keeps a long distance from the electrode. These results indicate that the thiol-terminated aptamer has been successfully immobilized onto the electrode.

### Electrochemical Detection of PCB77 by the RGO-AuNP-Ap/Au Electrode

**Figure 3:**
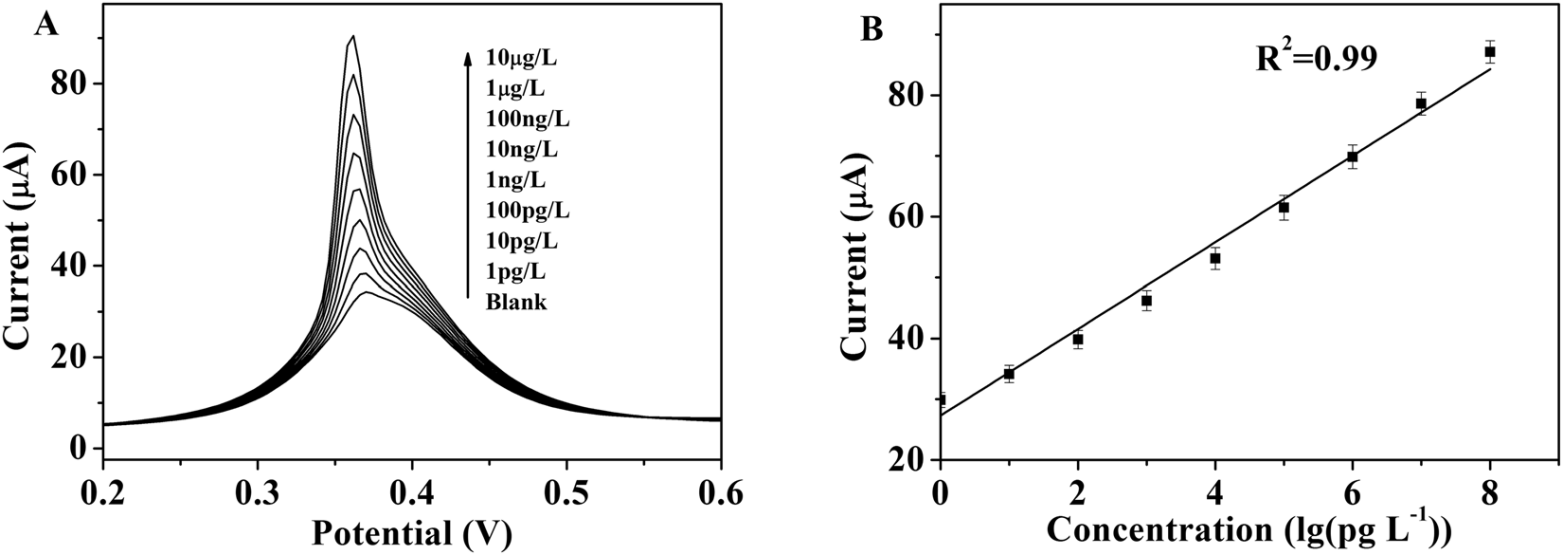
(**A**) Differential pulse voltammograms (DPV) of the RGO-AuNP-Aptamer/Au biosensor at different concentrations of PCB77 ranging from 1 pg L^−1^ to 10 µg L^−1^, respectively. Pulse amplitude, 50 mV; pulse period, 0.2 s. (**B**) The increase of the peak current for PCB77 at different concentrations. The standard deviations are all less than 5%.

PCB77 is one of the most important carcinogen and environmental pollutant and a type of structurally coplanar dioxin-like PCB with strong toxicity. Its environmental behavior and biological toxicology have been extensively studied^17, 18^. Because of its bioaccumulation and immunotoxic effect, Danis’ group suggested that PCB77 should be included in the list of marine monitoring programs^19^. Based on the above reasons, PCB77 is chosen as the target among these PCBs in this research. As a sensitive method, DPV is used for monitoring the concentration of PCB77 by the RGO-AuNP-Ap/Au biosensor. The interaction between PCB77 and aptamer is electrochemically magnified by redox-active ferrocene molecules. As shown in Fig. 3, the response signal of this biosensor is successively improved by the addition of PCB77. As shown in Fig. 3A, the addition of PCB77 (concentration ranging from 1 pg L^−1^ to 10 µg L^−1^) successively increased the response signal of RGO-AuNP-Ap/Au biosensor. The peak currents of the RGO-AuNP-Ap/Au biosensor exhibited a linear relationship with the PCB77 concentration ranging from 1 pg L^−1^ to 10 µg L^−1^ (Fig. 3B). The detection limit for PCB77 is as low as 0.1 pg L^−1^. Compared with the LODs obtained using previously reported methods, the LOD of this biosensor (0.1 pg L^−1^) has significantly surpassed that of both the electrochemical Aptamer-MWNT/GC biosensor (3.42 μg L^−1^)^20^ and our previous reported (Aptamer/Au biosensor (0.01 μg L^−1^)^21^.

In addition, the performances of the RGO-AuNP-Ap/Au biosensor, the AuNP-Ap/Au biosensor and Ap/Au biosensor are comparatively and systemically studied. The sensitivity of the RGO-AuNP-Ap/Au biosensor, the AuNP-Ap/Au biosensor and the Ap/Au biosensor are 226.8, 147.2 and 1.07 μA cm^−2^, respectively. Reproducibility and stability are the other important characters of the RGO-AuNP-Ap/Au biosensors. The inter-electrode relative standard deviation (RSD) of the RGO-AuNP-Ap/Au biosensor is below 5%. The long-term stability of this biosensor is evaluated by DPV in the presence of 100 ng L^−1^ PCB77. After two weeks of refrigerated storage at 4 °C, this biosensor still retains over 85% of its initial response signal.

**Figure 4:**
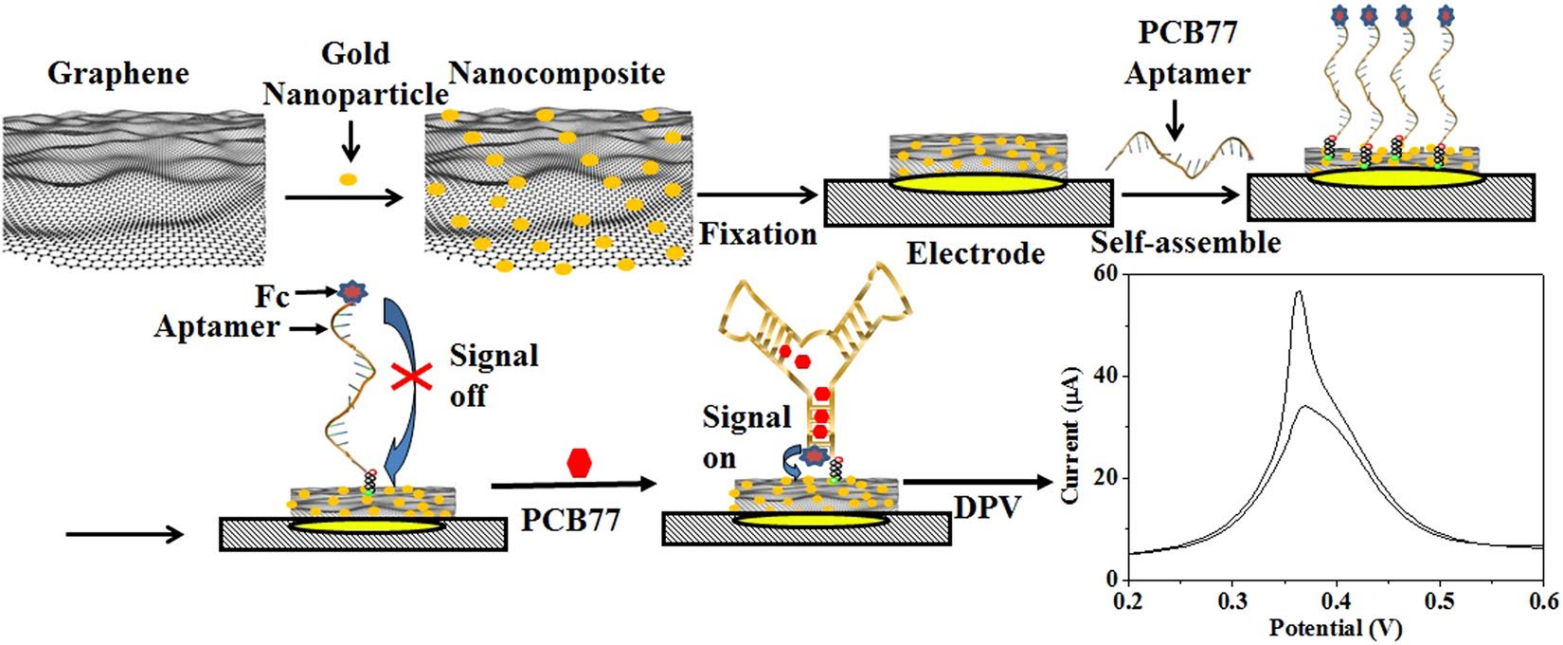
Schematic diagram of the fabrication process and detection mechanism of the RGO-AuNP-Ap/Au biosensor.

The super sensing performance of RGO-AuNP-Ap/Au biosensor may be ascribed to the following factors: First, the large specific surface area of RGO and gold nanoparticles could significantly increase the active surface area of the Au electrode to self-assemble aptamer. Second, the superior electric conductivity of RGO and gold nanoparticles can improve the electron transfer rate between the surface of Au electrode and ferrocene molecules. Third, as shown in Fig. 4, in the absence of PCB77, the aptamers are believed to remain unfolded and the detection response signal remains low. Upon PCB77 binding, the aptamer is folded into a configuration that forces the ferrocene molecules modified on the aptamer to be in the proximity of the aptamer/Au sensor^22, 23^, and the response signal increases. Sun’s group used Raman and SERS to confirm that the aptamer/Au sensor are more likely to form a hairpin loop^23^. Our conclusion is consistent with theirs’ conclusion. Fourth, the ferrocene molecules may change into ferrocenium on the electrochemical biosensor, and subsequently it may be recovered by the aqueous potassium ferricyanide solution, so it can provide a stable electrochemical amplification signal of ferrocene molecule^24^. Overall due to the above mentioned unique features, the RGO-AuNP-Ap/Au biosensor exhibits high sensitivity and low LOD. Thus, the RGO-AuNP-Ap/Au biosensor displays greater potential for use as a “pre-alarm” tool on site monitoring the PCBs pollutants.

### Background Interferences and Tapwater Samples Monitoring by the RGO-AuNP-Ap/Au Electrode

**Figure 5:**
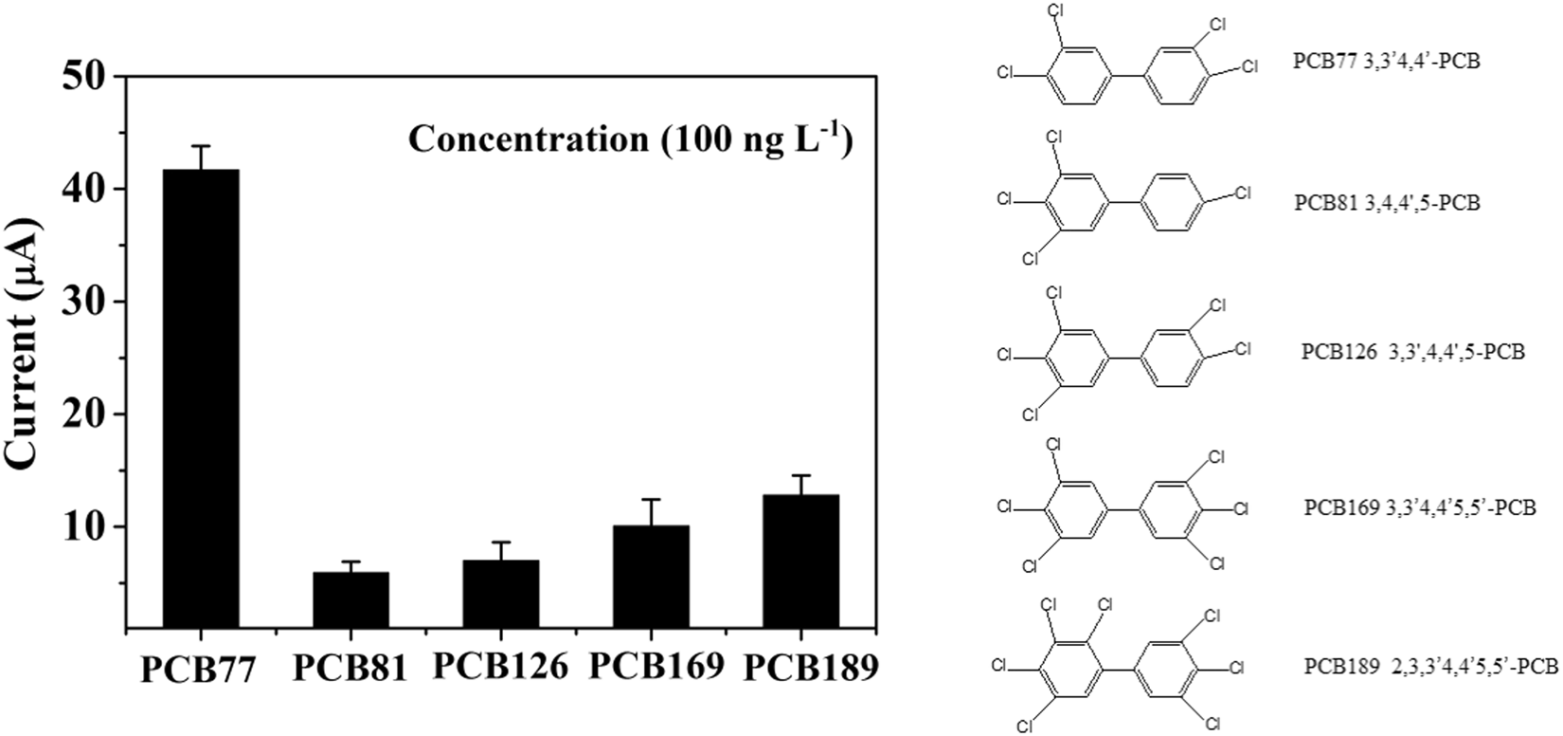
Screening of five co-planar PCBs (i.e., PCB77, PCB81, PCB126, PCB169 and PCB189) using the RGO-AuNP-Ap/Au biosensor (in the left figure), the illustrative structural diagrams of PCB77, PCB81, PCB126, PCB169 and PCB189 (in the right figure).

Co-planar PCBs (e.g., PCB77, PCB81, PCB126 and PCB189) are a family of halogenated persistent toxic chemicals, the endocrine disruptive effects of PCBs and persistent environmental pollutants that elicit a number of adverse health effects including teratogenesis, immunotoxicity, neurotoxicity, reproductive toxicity and carcinogenesis. The selective character of developed RGO-AuNP-Ap/Au biosensor is assessed by the other co-planar PCBs and benzene derivatives. Figure 5 shows the DPV response signals of the RGO-AuNP-Ap/Au biosensor for screening of 5 types of target analytes at 100 ng L^−1^. Because the aptamer is selectively enriched from PCB77 using SELEX technology, the response signal of PCB77 exhibits the largest change among these chemicals. This biosensor is also used for screening benzene derivatives (e.g., chlorobenzene and hexachlorobenzene), because these chemicals always coexist with PCBs and have similar physicochemical properties. These benzene derivatives do not produce any signal changes in the research, so the data does not display in the Fig. 5.

In addition, tap water samples are used for evaluating the feasibility of the RGO-AuNP-Ap/Au biosensor. Tap water spiked with different PCB77 concentrations (i.e., 20, 60, 100 ng L^−1^ PCB77) is analyzed using the RGO-AuNP-Ap/Au biosensor and GC-MS. The results indicate that the difference in the values are −9.76%, 7.75% and −7.3%, respectively, which reveals that the developed biosensor is a potential and efficient tool for the selective differentiation of PCB77 from coexisting chemicals.

## Methods

### Materials and Solutions

3,3′4,4′-PCB (PCB77), 3,4,4′,5-PCB (PCB81), 3,3′,4,4′,5-PCB (PCB126), 3,3′4,4′5,5′-PCB (PCB169), 2,3,3′4,4′5,5′-PCB (PCB189) are obtained from Dr. Ehrenstorfer (Germany). Other reagents are obtained from Sigma Aldrich (USA). PCB77 and other PCB congeners are dissolved in the solution of H_2_O and dimethyl formamide (V:V, 4:1) to get series of standard solutions, respectively. The aptamer probe (developed by SELEX^25^) 5′-SH-(CH_2_)_6_-GGCGGGGCTACGAAGTAGTGATTTTTTCCGATGGCCCGTG-Fc-3′ is prepared by Takara Biotechnology Co. Ltd. (Dalian, China) and dissolved in 20 mmol L^−1^ Tris-HCl (pH 8.0) containing 100 mmol L^−1^ MgCl_2_. Unless otherwise mentioned, 5 mmol L^−1^ potassium ferricyanide and potassium ferrocyanide [Fe(CN)_6_]^4−/3−^ (named as “FC solution”) is used as the electrolyte.

### Instrumentations

Transmission electron microscopy (TEM) images are obtained by a JEM-2100 (Japan). UV-Vis curve is gotten by a UV-Vis spectroscopy (Germany). The zeta potential of RGO and gold nanoparticles are measured by Zetasizer (Malvern, UK). X-ray photoelectron spectroscopy is tested by Thermo-VG Scientific (USA). A CHI 660B Electrochemical Workstation (CHI Instruments Inc.) is used for differential pulse voltammetry (DPV) and cyclic voltammetry (CV) tests. The three-electrode system consists of an Au electrode as the working electrode, an Ag/AgCl electrode as the reference electrode, and a platinum wire as the auxiliary electrode.

### Preparation of RGO and RGO-AuNP

The RGO is prepared by a ball milling method according to our previous report^26^. In this experiment, 2.0 g graphite powder and 60 g steel balls (diameter: 1 cm) are put into a hardened steel vial inside a glove box and this vial is purged with high purity argon (99.999%) for 20 min before being sealed. The ball milling is carried out at 450 rpm for 20 hours to yield graphene oxide. Subsequently, 0.5 mg mL^−1^ graphene oxide solution (50 mL) is adjusted to pH value 10 by NaOH solution (8 mol L^−1^) and added 29 mg Vitamin C (Vc) into the solution at 80 °C for 24 hours. This solution is centrifuged at 13000 rpm for 5 minutes and then removed the liquid supernatant for three times. Finally, the chemically reduced graphene oxide (RGO) is obtained.

Preparation of gold nanoparticles by seed-induced growth method: gold nanoparticles are produced in liquid by reduction of chloroauric acid. 50 mL 0.1 mg mL^−1^ chloroauric acid is rapidly stirred for 2 minutes after 2.5 mL Vitamin C (4 mg mL^−1^) added. And then 0.5 mL sodium citrate (10 mg mL^−1^) is added into the above solution to cease the chemical reaction. Through the centrifugation (13000 rpm, 5 minutes) for three times, the gold nanoparticles (AuNP) are obtained.

1.5 mL RGO and 0.5 mL AuNP are mixed and stirred for 30 minutes, and then the above process repeats 5 times. Finally, the composites (RGO-AuNP) are obtained.

### Preparation of the RGO-AuNP-Ap/Au electrode

The RGO-AuNP-Ap/Au electrode is prepared as follows: prior to modification, the surface of the Au electrode is polished by series of alumina (0.3 and 0.05 µm diameter) and then ultrasonic washing with ethanol and deionized water for three times, respectively. Then, the Au electrode electrochemically cleaned by potential scanning in 1 mol L^−1^ H_2_SO_4_ from 0 to 1.7 V until reproducible cyclic voltammograms are gotten. The preparation process involves shaking the solution of RGO-AuNP nanomaterials (0.5 mg mL^−1^) by ultrasound wave for 20 minutes and then 6 µL this solution is added onto the electrode surface. Then, the thiolated aptamer solution (8 µL, 1 µmol L^−1^) is dropped onto the RGO-AuNP modified Au electrode for 6 hours at room temperature to get the RGO-AuNP-Ap/Au electrode.

Before electrochemical measurements, all of the electrodes are washed with excess deionized water to remove the aptamer that are not adsorbed. The other electrodes are prepared following similar protocol as designed for the RGO-AuNP-Ap/Au electrode. Further, the RGO-AuNP-Ap/Au electrode and other electrodes are characterized by electrochemical scanning in a 5 mmol L^−1^ FC solution.

### Detection of PCB77 with RGO-AuNP-Ap/Au

Among these PCBs congeners, PCB77 which is a structurally coplanar and dioxin-like compound may cause cancer. Using PCB77 as a target compound, 8 µL PCB77 solution is pipetted into the FC solution for 5 minutes to provide enough time for using aptamer to combine PCB77 sufficiently. Subsequently, DPV method is carried on the RGO-AuNP-Ap/Au biosensor in the FC solution to monitor the response signal changes produced by PCB77. More than 6 kinds of chemicals (100 ng L^−1^) are also detected by the RGO-AuNP-Ap/Au biosensor including PCBs congeners (PCB81, 126, 169 and 189) or benzene derivatives (chlorobenzene, pentachlorobenzene, etc.).

### Safety Considerations

PCBs and the benzene derivative (chlorobenzene, pentachlorobenzene, etc.) are carcinogenic agents. MSDS information for these chemicals must be consulted, and standard laboratory protocols must be strictly followed before handling the chemicals.

## Electronic supplementary material

supporting information
