# Supplementary material for: Gold Nanoparticles dotted Reduction Graphene Oxide Nanocomposite Based Electrochemical Aptasensor for Selective, Rapid, Sensitive and Congener-Specific PCB77 Detection
Source: Sci Rep. 2017 Jul 12;7:5191. doi: 10.1038/s41598-017-05352-7 (PMC5507977; doi:10.1038/s41598-017-05352-7)
Supplement: Supplementary file 1 — supporting information [file 41598_2017_5352_MOESM1_ESM.doc]

**Supporting Information**

Gold Nanoparticles dotted Reduction Graphene Oxide Nanocomposite Based Electrochemical Aptasensor for Selective, Rapid, Sensitive and Congener-Specific PCB77 Detection

Lidong Wu1[[1]](#footnote-2)*, Xianbo Lu^2*^, Xiaochen Fu^1^, Lingxia Wu^2^, Huan Liu^1^

^1^Chinese Academy of Fishery Sciences, Beijing 100141, China

^2^Key Laboratory of Separation Science for Analytical Chemistry, Dalian Institute of Chemical Physics, Chinese Academy of Sciences, Dalian 116023, China

Figure S1. The UV-Vis absorption spectrum of the RGO-AuNP nanocomposites.
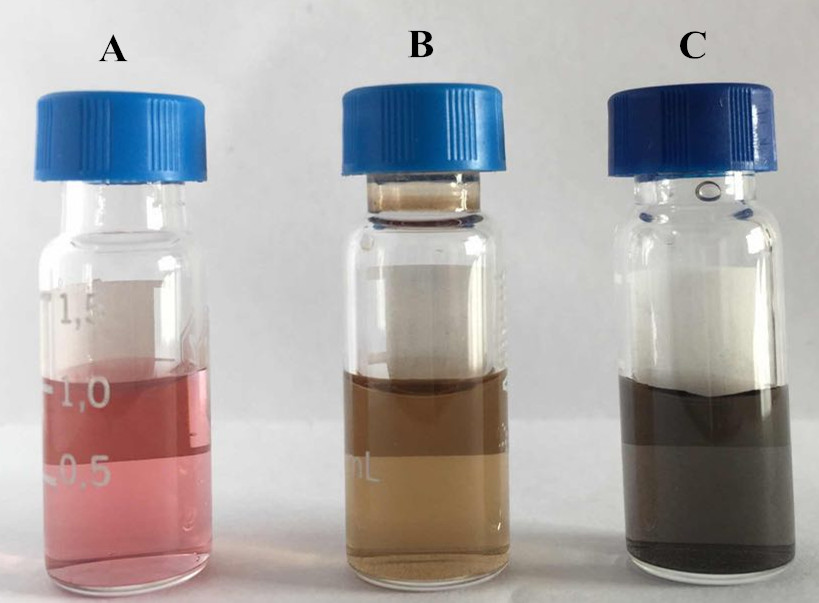


Figure S2. Photographs of dispersions of 0.4 mg ml^-1^ AuNPs (A), 0.4 mg ml^-1^ RGO (B) and 0.4 mg ml^-1^ RGO-AuNPs (C) in aqueous solutions.


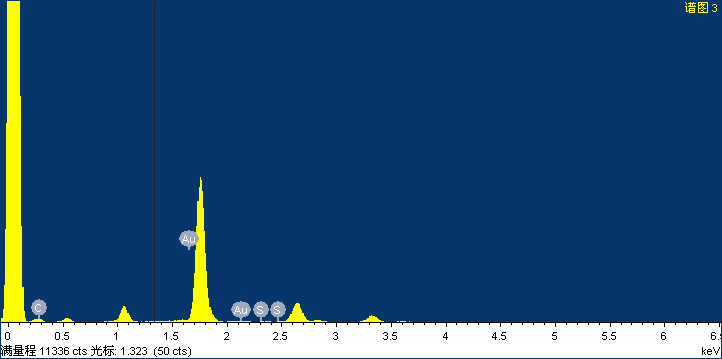


Figure S3. X-ray photoelectron spectroscopy of RGO-AuNPs.

1. ^^ Corresponding author. Tel/Fax: +86-10-68690712; E-mail: [lidongwu@mit.edu](mailto:lidongwu@mit.edu) [↑](#footnote-ref-2)
